# Comparison of the number of oocytes obtained after ovarian stimulation between Chinese and Caucasian women undergoing in vitro fertilization using a standardized stimulation regime

**DOI:** 10.1186/s13048-021-00928-4

**Published:** 2021-12-11

**Authors:** Jennifer K. Y. Ko, Andrew Kan, Peter Leung, Vivian C. Y. Lee, Raymond H. W. Li, William Ledger, Ernest H. Y. Ng

**Affiliations:** 1 Department of Obstetrics and Gynecology, The University of Hong Kong, Queen Mary Hospital, Hong Kong, China; 2 IVF Australia, Sydney, Australia; 3 Discipline of Obstetrics and Gynecology, School of Women’s and Children’s Health, University of New South Wales, Sydney, Australia; 4 Department of Obstetrics and Gynecology, The University of Sydney, Sydney, Australia; 5 Premier Medical Centre, Hong Kong, China

**Keywords:** ethnicity, ovarian response, in vitro fertilization, infertility, corifollitropin alfa

## Abstract

**Background:**

In vitro fertilization (IVF) is a well-established method to treat various causes of infertility. Some previous retrospective studies suggested a lower ovarian response in Asian women compared to Caucasian women. However, the ovarian stimulation regimens were not standardized, potentially confounding the findings. The objective of this study is to compare the number of oocytes obtained after ovarian stimulation between Chinese and Caucasian women undergoing IVF using a standardized stimulation regimen.

**Methods:**

This is a prospective cohort study conducted in two tertiary IVF units in Hong Kong, China and Sydney, Australia from October 2016 to August 2019. A total of 192 women aged 18–42 years with a body weight > 60 kg underwent IVF with a standard ovarian stimulation regimen of 150 micrograms corifollitropin alfa (Elonva®) followed by 200 IU follitropin beta (Puregon®) per day. The number of oocytes retrieved in Chinese women treated in the Hong Kong center was compared to that of Caucasian women treated in the Australian center.

**Results:**

Serum AMH levels were similar between the two groups. Although women in the Chinese cohort were older and had a higher body mass index (BMI), longer duration of infertility and lower antral follicle count (AFC) than those in the Caucasian cohort in this study, no differences in the number of oocytes retrieved [11 (8–17) vs. 11 (6–17), p=0.29], total dosage and duration of stimulation and number of follicles aspirated were noted between the two ethnic cohorts. The peak estradiol level was greater in Chinese women than in Caucasian women. After controlling for age, BMI and AFC, ethnicity was a significant independent determinant of the number of oocytes obtained.

**Conclusions:**

Chinese women had a higher number of oocytes after ovarian stimulation using a standardized stimulation regimen compared with Caucasian women undergoing IVF after controlling for age, BMI, AFC and AMH despite presenting later after a longer duration of infertility. Trial registration number: NCT02748278

## Introduction

In vitro fertilization (IVF) is a well-established method to treat various causes of infertility. Many factors can affect its outcome. In women, these factors include a woman’s age [1–3], body mass index [4], duration and type of infertility [1–3], previous live birth [3], number of previous unsuccessful IVF cycles [3], smoking status [5] and ovarian reserve [6], which subsequently affect parameters in the IVF cycle, including the number of oocytes retrieved [2, 7, 8] and embryo quality [2], to determine whether an IVF cycle is likely to be successful. Evidence that ethnic variation in IVF outcomes exists comes from large national registries in the United States and the United Kingdom, which showed poorer IVF outcomes in Asian, African and Hispanic women compared with Caucasian women [9–12]. These registry studies include data from various assisted reproductive centers with different population mixes, treatment regimens and success rates and could include the same woman who underwent multiple IVF cycles during the study period, thereby introducing confounding factors and bias. Specific clinical studies have reported similar findings of lower clinical pregnancy and live birth rates in Asian women compared with Caucasian women [13–15]. However, in one study, these findings were not significant after controlling for age and duration of infertility because Asian women were older and sought fertility treatment later [13].

In many of these programs, Caucasians represented the majority of the study population and were taken as the ‘reference group’. Different ovarian stimulation protocols might be used even within the same clinic potentially due to clinician preconceptions about the likely response of different ethnic groups to FSH, thereby affecting their patient’s IVF outcome. Moreover, the use of ‘Asian’ as an ethnic category can be misleading as this group includes women of ethnic groups with different genetic backgrounds from all parts of the largest continent of the world, making the results difficult to interpret.

All published studies on this topic are retrospective, and no prospective study comparing ovarian response and reproductive outcomes of superovulation for IVF between different ethnic groups has been reported. The objective of our study was to compare the number of oocytes obtained after ovarian stimulation using a standardized stimulation regime between Chinese and Caucasian women undergoing IVF.

## Materials and methods

We conducted a prospective cohort study comparing ovarian response in Chinese and Caucasian women in two tertiary IVF units in Hong Kong, China and Sydney, Australia respectively, from October 2016 to August 2019. The standardized IVF protocol was agreed upon at a meeting of the investigators from the two units before the start of the study.

### Study population

Infertile women undergoing IVF in these two units were recruited if they were aged 18–42 years old and had body weight >60 kg and total antral follicle count (AFC) between 7 and 20. Ethnicity was self-reported. Caucasians were defined as originating from the United Kingdom, Europe or the United States of America, excluding the Middle East. Women were excluded if they were of mixed race, had a history of ovarian surgery, had a body mass index >35 kg/m^2^, had more than 2 previous stimulated IVF cycles or had ovarian hyperstimulation syndrome in previous stimulated IVF cycles. Written consent was obtained from all participants. The study was approved by the Institutional Review Board of the University of Hong Kong/Hospital Authority Hong Kong West Cluster (UW 15–547) and IVF Australia Ethics Committee (IRB number 116). The study was registered at clinicaltrials.gov (trial registration number: NCT02748278).

### Ovarian stimulation and IVF

IVF was performed in the two units with a standardized stimulation regimen of 150 micrograms corifollitropin alfa (Elonva®) followed by 200 IU follitropin beta (Puregon®) per day. The details of the IVF techniques in the two centers were previously published [13, 16]. Women attended the clinic for an ultrasound scan on the second or third day (Day 2 or 3) of their period to exclude the presence of ovarian cysts and had their AFC determined, which included all follicles of 2–10 mm measured with a 7.0–9.0 MHz transvaginal ultrasound probe. Serum anti-Mullerian hormone (AMH) levels were assessed on the same day (Day 2 or 3) and analyzed using the Elecsys® AMH assay (Roche Diagnostics, Mannheim, Germany). Ovarian stimulation commenced if no ovarian cyst was found on ultrasound scan. Women received one injection of 150 micrograms of long-acting gonadotrophin corifollitropin alfa (Elonva®, NV Organon, Oss, The Netherlands) subcutaneously followed by daily 200 IU gonadotrophin injections of follitropin beta (Puregon®, NV Organon, Oss, The Netherlands) starting 7 days after the Elonva® injection in an antagonist protocol. Then, 0.25 mg GnRH antagonist ganirelix acetate (Orgalutran®, NV Organon, Oss, The Netherlands) was started on Day 5 of ovarian stimulation. Transvaginal ultrasound scans were performed for follicular tracking 7 days after the Elonva® injection and every 1–3 days thereafter, depending on the ovarian response. FSH dose adjustment was not allowed. Cycles were cancelled if there were less than 3 follicles larger than 18 mm or no developing follicle (i.e., larger than 11 mm) after one week of 200 IU Puregon®.

Next, 0.25 mg recombinant hCG (Ovidrel®, Serono, Bari, Italy) was administered when 2 follicles >18 mm in diameter were present. Gonadotrophin injection was not administered on the day of the ovulatory trigger. An agonist trigger was used if the serum estradiol level on the day of trigger was greater than 15,000 pmol/L or if there were greater than 15 follicles larger than 16 mm on transvaginal scanning. All embryos or blastocysts were frozen for transfer later following the agonist trigger or when 20 or more oocytes were aspirated. Elective freezing was also considered if the woman had premature progesterone elevation, untreated hydrosalpinx or endometrial polyp or other personal circumstances in which fresh-embryo transfer was not preferred. Serum FSH, estradiol, LH and progesterone levels were measured 7 days after Elonva® injection and on the trigger day. Transvaginal ultrasound-guided oocyte retrieval (TUGOR) was scheduled 34–36 hours after the trigger injection. All follicles greater than 10 mm were aspirated. Flushing of follicles was not performed.

The retrieved oocytes were inseminated conventionally or by intracytoplasmic sperm injection (ICSI) depending on the semen parameters. One to two embryos were replaced on Days 2–5 after oocyte retrieval under transabdominal ultrasound guidance using a soft catheter. Luteal phase support was started according to the standard protocol of the unit: Crinone® 8% (90 mg) vaginal progesterone gel twice per day for 2 weeks in the Sydney center and Endometrin® (100 mg) vaginal progesterone insert twice per day for 2 weeks in the Hong Kong center. The remaining embryos or blastocysts were frozen.

### Follow-up

A urine pregnancy test was performed 18 days after the ovulatory trigger. If the pregnancy test was positive, transvaginal ultrasonography was performed two and four weeks later to confirm fetal viability. Women were referred for antenatal care when the pregnancy was ongoing at 8–10 weeks. Pregnancy outcomes were traced from the electronic patient record system or self-returned reply slips from the women or their obstetricians. If no reply letter was received 2–3 months after the expected date of confinement, the women were contacted by our nurses to trace the obstetric outcomes.

### Study outcome

The primary outcome was the number of oocytes retrieved. Secondary outcomes included the duration of stimulation and total dosage of FSH consumed, serum estradiol levels on day of ovulatory trigger, miscarriage rate, clinical pregnancy rate (presence of intrauterine gestational sac at 6 weeks on ultrasonography), ongoing pregnancy rate (presence of fetal heart pulsation on ultrasonography beyond 8 weeks), live birth rate beyond 22 weeks of gestation and rate of ovarian hyperstimulation syndrome.

### Statistical analysis

The average number of oocytes obtained in the two participating units was 12.0 with a standard deviation of 7.0. With the assumption that a difference of 3 oocytes between the two groups would be significant, the sample size required for a power of 0.8 and type I error of 0.05 was 174 women (87 in each group). To account for 10% loss to follow-up, 192 women (i.e., 96 in each group) were recruited.

Data were analyzed using IBM SPSS software (SPSS 25.0, IBM Corporation, NY, USA). Demographic characteristics and study outcomes of the two study groups were compared using the Mann–Whitney test and chi-squared test for continuous and categorical variables, respectively. Multiple linear regression analysis was used to determine the relationship of ethnicity with the number of oocytes retrieved. A two-tailed value of P<0.05 was considered statistically significant.

## Results

**Fig. 1:**
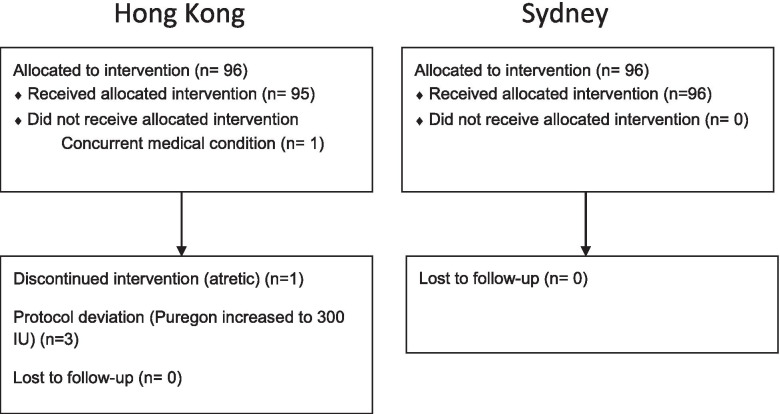
Flow chart of participants

A total of 192 women (96 from each unit) were recruited (Figure 1). In the Chinese cohort, one woman withdrew from the study before IVF due to concurrent medical problems, and the IVF cycle was cancelled for one woman due to atretic follicles and vaginal bleeding during ovarian stimulation.

The demographic characteristics are shown in Table 1. Women in the Chinese cohort were older and had a higher BMI and longer duration of infertility than those in the Caucasian cohort in this study. Chinese women had a lower AFC than Caucasian women in this study, but both Chinese and Caucasian women had similar serum AMH levels.

**Table 1:** Demographic characteristics of participants

	Chinese (n=96)	Caucasians (n=96)	P value
Age of women (years)	37 (35-38)	35 (32-37)	<0.01
Height of women (cm)	162.0 (157.3-165.3)	165.0 (162.0-170.0)	<0.01
Body mass index (kg/m^2^)	25.2 (23.6-27.3)	23.8 (22.3-26.7)	0.02
Primary infertility	64 (66.7%)	60 (62.5%)	0.34
Cause of infertility			<0.01
Endometriosis Male Tubal Unexplained Mixed Anovulation Same sex Donor sperm Fertility cryopreservation PGT	4 (4.2%) 43 (44.8%) 11 (11.4%) 24 (25.0%) 10 (10.4%) 4 (4.2%) 0 (0%) 0 (0%) 0 (0%) 0 (0%)	3 (3.1%) 25 (26.0%) 2 (2.1%) 39 (40.6%) 3 (3.1%) 0 (0%) 9 (9.4%) 7 (7.3%) 4 (4.2%) 4 (4.2%)	
Duration of infertility (years)	4.2 (3.0-6.0)	1.8 (1.0-2.0)	<0.01
Previous IVF cycles before	23 (25.0%)	27 (28.1%)	0.62
Non-smoker/ ex-smoker (quitted at least 1 year)	84 (87.5%)	91 (94.8%)	0.07
Regular cycles	85 (88.5%)	88 (91.7%)	0.38
Antral follicle count	11 (8-14)	14 (10-17)	0.03
AMH (pmol/L)	14.7 (10.0- 20.3)	11.8 (6.3- 22.7)	0.33

Data presented as median (25^th^ -75^th^ percentile or number (percentage)

PGT – preimplantation genetic testing

AFC- antral follicle count, AMH- anti-mullerian hormone

No differences in the number of oocytes retrieved [11 (8–17) vs. 11 (6–17), p=0.29], total dosage and duration of stimulation or total number of follicles aspirated were noted between the two ethnic cohorts (Table 2). The peak estradiol level was higher in Chinese women compared with Caucasian women [8538 (5861–12206) vs. 6501 (3676–9495) pmol/l, p<0.01].

**Table 2:** IVF stimulation characteristics

	Chinese (n=94)*	Caucasians (n=96)	P value
Duration of stimulation (days)	10 (10-12)	10 (9-11)	0.10
Dosage of FSH used in addition to Elonva® (IU)	600 (600-1000)	800 (450-1500)	0.12
Serum estradiol level at day 9	2733 (1652-4813)	2762 (1690-4909)	0.76
Serum estradiol level on the day of trigger (pmol/L)	8535 (5861-12206)	6501 (3676-9495)	<0.01
Serum progesterone level on the day of trigger (nmol/L)^	3.3 (2.2-4.1)	2.3 (1.6-3.0)	<0.01
Agonist trigger	9 (9.6%)	31 (32.3%)	<0.01
Endometrial thickness (mm)	11.3 (10.0-13.6)	9.0 (8.0-10.0)	<0.01
Number of follicles aspirated	14 (10-24)	13 (9-20)	0.22
Number of oocytes collected	11 (8-17)	11 (6-17)	0.29
Proportion of ICSI	38/94 (40.4%)	59/96 (61.5%)	<0.01
Number of oocytes fertilized	9 (5-13)	5 (3-9)	<0.01
Fresh embryo transfer	66/94 (70.2%)	42/96 (43.8%)	<0.01
Number of cleavage embryos transferred			0.09
1 2	19 (50%) 19 (50%)	3 (100%) 0 (0%)	
Number of blastocysts transferred			0.58
1 2	25 (89.3%) 3 (10.7%)	33 (84.6%) 6 (15.4%)	
Blastocyst culture	45/94 (47.9%)	86/96 (89.6%)	<0.01
Blastocyst transfer	28/66 (42.4%)	39/42 (92.9%)	<0.01
OHSS (early onset)^#^	5 (5.3%)	2 (2.1%)	0.236

Data presented as median (25^th^ -75^th^ percentile) or number (percentage)

hCG- human chorionic gonadotrophin

*1 cancelled before IVF started due to concurrent illness, 1 cancelled IVF cycle because of vaginal bleeding and atretic follicles

#all mild

^ for those with fresh transfer only

No significant difference in the ongoing pregnancy rates per fresh transfer were noted between the Chinese and Caucasian groups; however, a higher proportion of blastocyst transfer was observed in the Caucasian group (Table 3). One Chinese woman and 13 Caucasian women did not have embryos for transfer or freezing.

**Table 3:** Pregnancy outcome per fresh transfer

	Chinese	Caucasians	*P* value
Positive pregnancy test			
Overall Blastocyst	23/66 (34.8%) 11/28 (39.3%)	19/42 (45.2%) 19/39 (48.7%)	0.28 0.44
Clinical pregnancy			
Overall Blastocyst	20/66 (30.3%) 8/28 (28.6%)	15/42 (35.7%) 15/39 (38.5%)	0.56 0.40
Ongoing pregnancy			
Overall Blastocyst	17/66 (25.8%) 6/28 (21.4%)	14/42 (33.3%) 14/39 (35.9%)	0.40 0.20
Live birth			
Overall Blastocyst	15/66 (22.7%) 5/28 (17.9%)	11/42 (26.2%) 11/39 (28.2%)	0.68 0.33
No fresh transfer	28/94 (29.8%)	53/96 (54.2%)	<0.01

After controlling for age, BMI and AFC or AMH in a multiple linear regression model using the standard method, ethnicity was identified as a significant independent determinant of the number of oocytes obtained. In addition, AFC (Table 4a) and AMH (Table 4b) were also significant independent determinants, whereas BMI was not.

**Table 4:** Regression analysis of factors predicting the number of oocytes retrieved

	Unstandardized B (95% CI)	P value
(a) Controlling for age, body mass index, ethnicity and antral follicle count		
Age of women (years)	-0.8 (-1.2 to -0.5)	<0.01
Body mass index	-0.2 (-0.5 to -1.5)	0.29
Ethnicity	-5.6 (-7.7 to -3.5)	<0.01
Antral follicle count	0.7 (0.5 to 1.0)	<0.01
(b)Controlling for age, body mass index, ethnicity and anti-Mullerian hormone		
Age of women (years)	-0.5 (-0.8 to -0.2)	<0.01
Body mass index	-0.2 (-0.4 to 0.2)	0.07
Ethnicity	-2.5 (-4.1 to -0.9)	<0.01
AMH	0.4 (0.4 to 0.5)	<0.01

When women undergoing IVF in a same-sex relationship or women using donor sperm, fertility cryopreservation and preimplantation genetic testing and the 3 Chinese women who experienced protocol violations (required stepping up of Puregon® dosage) were excluded, Chinese women remained significantly older, had a longer duration of infertility and had a higher number of oocytes retrieved despite no significant difference in AMH when compared with Caucasian women (Table 5).

**Table 5:** Excluding same-sex relationship/ single women using donor sperm, fertility preservation, PGT and protocol violation

	Chinese (n=91)	Caucasians (n=72)	P value
Age of women	37 (35-38)	36 (33-37)	<0.01
BMI	25.2 (23.4-27.1)	23.7 (22.4-26.6)	0.04
Antral follicle count	11 (8-14)	14 (10-17)	0.03
Duration of infertility	4.0 (3.0-6.0)	2.0 (1.0 -2.5)	<0.01
Number of follicles aspirated	14 (10-22)	12 (8-17)	0.04
Number of oocytes retrieved	11 (8-17)	10 (5.5-13.5)	0.04

## Discussion

Our study showed no evidence of a reduction in the number of oocytes obtained after a standard stimulation regimen using 150 micrograms corifollitropin alfa followed by a fixed dose of follitropin beta in Chinese compared with Caucasian women despite the fact that the Chinese women were older. Ethnicity remained a significant determinant of the number of oocytes obtained after controlling for the age of women, BMI, AFC and AMH, suggesting that Chinese ethnicity was associated with a better ovarian response than that noted in Caucasian women. This feature was not accompanied by a better pregnancy rate, ongoing pregnancy rate or live birth rate compared with Caucasian women, which can partially be explained by the older age of the Chinese women in this cohort. However, interpretation of the pregnancy outcomes (secondary outcomes) is limited by the presence of confounding factors in the study groups.

Previous studies have generally suggested a lower live birth rate in Asian populations compared with Caucasians, but the definition of ‘Asian’ has varied considerably between publications [11, 12, 14]. Published data from the nationwide program in the United Kingdom did not show fewer oocytes retrieved or lower live birth rates after IVF in British Chinese women compared with white British women, but British Chinese women had a significantly lower mean number of embryos stored even though they were not significantly older [9]. In a clinic-focused study by the same group in the United Kingdom, their nonwhite population, including South-Eastern and Middle-Eastern Asians, had significantly poorer IVF outcomes despite having more favorable pretreatment ovarian reserve variables, including age, basal FSH, AFC, and similar ovarian response, fertilization and cleavage rates, suggesting a reduction in implantation [17]. Another study estimated the effect of ethnicity on IVF after blastocyst transfer in Asians and Caucasians in an attempt to eliminate the impact of embryo quality but still found lower clinical pregnancy and live birth rates in Asian women compared with Caucasian women, supporting a difference in endometrial receptivity [15]. In single-centered retrospective cohort studies, South Asian women had significantly lower live birth rates than Caucasian women after fresh embryo transfer but not following frozen-embryo transfer, suggesting endometrial rather than embryonic differences [18, 19]. South Asian women have also have a higher peak serum estradiol during ovarian stimulation than Caucasian women, even after adjusting for follicle number [20]. Similarly, we found a significantly higher peak serum estradiol level and number of oocytes retrieved in Chinese women after controlling for confounders. One possible explanation is that ethnic variation in steroidogenic profiles exists during ovarian stimulation, which subsequently affect endometrial receptivity.

Despite standardizing the stimulation regimen before the start of the study, variations in the case populations were noted in the two units, which reflected important differences in the health seeking practice of these populations. The Caucasian population included those undergoing IVF in a same-sex relationship, fertility cryopreservation and preimplantation genetic testing, which could potentially skew the population studied to be of younger age. IVF in a same-sex relationship is not permitted in Hong Kong. The rate of blastocyst transfer was much higher in the Caucasian group. This difference reflects differences in practice between the two units rather than a genuine difference in embryo quality. In the center in Hong Kong, women were advised to have their embryos cultured to blastocysts if they had 6 or more embryos on Day 2. In contrast, blastocyst transfer is the norm and performed in greater than 90% of cases in the Australian unit. More Caucasian women did not have fresh embryos available for transfer. However, these differences are unlikely to have affected the interpretation of the primary outcome, which was the number of oocytes retrieved. Our study was underpowered for the comparison of pregnancy and live birth rates, but reviewing the cumulative live birth rate may be informative.

Unexpectedly, Chinese women in our study had a higher BMI than Caucasians because the study recruited only women who weighed more than 60 kg to receive 150 micrograms of Elonva®. When controlling for body weight, Chinese women were generally shorter than their Caucasian counterpoints, resulting in a higher BMI. An increase in BMI may have a greater impact in Asian women than in Caucasian women. In one study, starting from a BMI of 25 kg/m^2^, Asian women had a lower live birth rate than Caucasian women [21].

Large database studies that have investigated the performance of ‘ethnic minorities’ in a ‘foreign’ country may have confounding factors, including differential access to treatment, differences in socioeconomic status between the groups under consideration, and the influences of lifestyle changes after migration. Women who have moved to another country may have different characteristics from those who remain in their country of origin. This phenomenon was illustrated in a study of immigrant Bangladeshi women, which found that those who grew up from childhood in the United Kingdom had significantly higher ovarian reserve markers than age-matched women who remained in Bangladesh or moved to the United Kingdom as adults [22]. Ethnicity was clearer in our setting, and socioeconomic problems were less likely to contribute to any differences in our study. Both units were tertiary reproductive units, and the study participants received care in their home country with good access to health care. However, ethnicity was self-reported in our study, which may have been a source of reporting bias. ‘Caucasians’ was also broadly defined in this study and encompassed individuals from at least two continents.

Despite the smaller sample size compared to the majority of the preceding retrospective studies on the topic, the strengths of our study are its prospective nature, its well-defined populations and the standardized stimulation regimen that was followed by women who participated in the study. Both units followed identical stimulation protocols. However, we only studied Chinese and Caucasian populations, so the results are not generalizable to other ethnic groups. We did not study the ethnicity of the partner; however, this factor was unlikely to affect the primary outcome. Other limitations of the study included the absence of matching the Chinese and Caucasian women based on age before the study, thereby making interpretations more difficult but controlling for use of the regression model. The study would be strengthened if the two groups were matched for age at the beginning of the study.

With ethnicity identified as a determinant factor in ovarian response, although it is not modifiable, measures can be taken to reduce the impact on the woman as we learn more about the ethnic variations in the different steps of IVF. Individualized ovarian stimulation regimens and embryo transfer protocols in centers catering to women of different ethnicities should take into account a woman’s ethnic background, especially in light of increasing global migration. Our results highlighted different health-seeking behaviors in different ethnic groups with the Chinese women in this study presenting later after a longer duration of infertility, confirming the findings of a previous retrospective study in the Australian unit [13]. A recent survey demonstrated a lack of fertility awareness among highly educated young Chinese people [23]. These individuals tended to overestimate success rates of fertility treatments and were less motivated to seek solutions in the event of fertility problems. Open discussion of fertility issues remains a cultural taboo. Measures should be taken to improve public education on fertility awareness to encourage Chinese women to start family planning early to maximize chances of achieving their desired family size. Although Chinese women had a higher number of oocytes obtained after ovarian stimulation using a standardized regimen than Caucasian women undergoing IVF after controlling for age, BMI, AFC and AMH, a more advanced age could lead to a suboptimal live birth rate. Further studies may investigate the ethnic differences in endometrial receptivity and focus on strategies to improve these differences.

## Conclusion

Chinese women had a higher number of oocytes obtained after ovarian stimulation using a standardized regimen than Caucasian women undergoing IVF after controlling for age, BMI, AFC and AMH.

## Data Availability

The data underlying this article will be shared on reasonable request to the corresponding author.

## References

[1] Bhattacharya S, Maheshwari A, Mollison J (2013). Factors associated with failed treatment: an analysis of 121,744 women embarking on their first IVF cycles. PLoS One..

[2] van Loendersloot LL, van Wely M, Limpens J, Bossuyt PM, Repping S, van der Veen F (2010). Predictive factors in in vitro fertilization (IVF): a systematic review and meta-analysis. Hum Reprod Update..

[3] Nelson SM, Lawlor DA (2011). Predicting live birth, preterm delivery, and low birth weight in infants born from in vitro fertilisation: a prospective study of 144,018 treatment cycles. PLoS Med..

[4] Sermondade N, Huberlant S, Bourhis-Lefebvre V, Arbo E, Gallot V, Colombani M (2019). Female obesity is negatively associated with live birth rate following IVF: a systematic review and meta-analysis. Hum Reprod Update..

[5] Waylen AL, Metwally M, Jones GL, Wilkinson AJ, Ledger WL (2009). Effects of cigarette smoking upon clinical outcomes of assisted reproduction: a meta-analysis. Hum Reprod Update..

[6] La Marca A, Sunkara SK (2014). Individualization of controlled ovarian stimulation in IVF using ovarian reserve markers: from theory to practice. Hum Reprod Update..

[7] Sunkara SK, Rittenberg V, Raine-Fenning N, Bhattacharya S, Zamora J, Coomarasamy A (2011). Association between the number of eggs and live birth in IVF treatment: an analysis of 400 135 treatment cycles. Hum Reprod..

[8] Polyzos NP, Drakopoulos P, Parra J, Pellicer A, Santos-Ribeiro S, Tournaye H (2018). Cumulative live birth rates according to the number of oocytes retrieved after the first ovarian stimulation for in vitro fertilization/intracytoplasmic sperm injection: a multicenter multinational analysis including approximately 15,000 women. Fertil Steril..

[9] Maalouf W, Maalouf W, Campbell B, Jayaprakasan K (2017). Effect of ethnicity on live birth rates after in vitro fertilisation/intracytoplasmic sperm injection treatment: analysis of UK national database. BJOG..

[10] Baker VL, Luke B, Brown MB, Alvero R, Frattarelli JL, Usadi R (2010). Multivariate analysis of factors affecting probability of pregnancy and live birth with in vitro fertilization: an analysis of the Society for Assisted Reproductive Technology Clinic Outcomes Reporting System. Fertil Steril..

[11] Fujimoto VY, Luke B, Brown MB, Jain T, Armstrong A, Grainger DA (2010). Racial and ethnic disparities in assisted reproductive technology outcomes in the United States. Fertil Steril..

[12] Purcell K, Schembri M, Frazier LM, Rall MJ, Shen S, Croughan M (2007). Asian ethnicity is associated with reduced pregnancy outcomes after assisted reproductive technology. Fertil Steril..

[13] Kan A, Leung P, Luo K, Fay L, Tan CL (2015). Do Asian women do as well as their Caucasian counterparts in IVF treatment: Cohort study. J Obstet Gynaecol Res..

[14] McQueen DB, Schufreider A, Lee SM, Feinberg EC, Uhler ML (2015). Racial disparities in in vitro fertilization outcomes. Fertil Steril..

[15] Langen ES, Shahine LK, Lamb JD, Lathi RB, Milki AA, Fujimoto VY (2010). Asian ethnicity and poor outcomes after in vitro fertilization blastocyst transfer. Obstet Gynecol..

[16] Li HWR, Ko JKY, Lee VCY, Yung SSF, Lau EYL, Yeung WSB (2020). Comparison of antral follicle count and serum anti Mullerian hormone level for determination of gonadotropin dosing in in-vitro fertilization: randomized trial. Ultrasound Obstet Gynecol..

[17] Jayaprakasan K, Pandian D, Hopkisson J, Campbell BK, Maalouf WE (2014). Effect of ethnicity on live birth rates after in vitro fertilisation or intracytoplasmic sperm injection treatment. BJOG..

[18] Mascarenhas M, Balen AH (2019). Could ethnicity have a different effect on fresh and frozen embryo transfer outcomes: a retrospective study. Reprod Biomed Online..

[19] Shah MS, Caballes M, Lathi RB, Baker VL, Westphal LM, Milki AA (2016). In vitro fertilization outcomes after fresh and frozen blastocyst transfer in South Asian compared with Caucasian women. Fertil Steril..

[20] Huddleston HG, Rosen MP, Lamb JD, Modan A, Cedars MI, Fujimoto VY (2010). Asian ethnicity in anonymous oocyte donors is associated with increased estradiol levels but comparable recipient pregnancy rates compared with Caucasians. Fertil Steril..

[21] Mascarenhas M, Kulkarni M, Balen A. Can the ethnic differences in IVF cycle outcome be influenced by the impact of BMI? Hum Fertil (Camb). 2019:1–7. 10.1080/14647273.2018.1563915 30642210

[22] Begum K, Muttukrishna S, Sievert LL, Sharmeen T, Murphy L, Chowdhury O (2016). Ethnicity or environment: effects of migration on ovarian reserve among Bangladeshi women in the United Kingdom. Fertil Steril..

[23] Chan CH, Chan TH, Peterson BD, Lampic C, Tam MY (2015). Intentions and attitudes towards parenthood and fertility awareness among Chinese university students in Hong Kong: a comparison with Western samples. Hum Reprod..

